# Carcinoma Uteri, Treatment

**Published:** 1874-04

**Authors:** 


The Treatment of Carcinoma Uteri.—R. Schroder gives the following details of the treatment he has adopted with success in cases of cancer of the uterus. After removing all the disease that was recognizable as a distinct tumor, a deep hollow was burnt with the actual cautery into the part corresponding with the cicatrix. The cautery was pressed with firmness into all those parts where new growth could be perceived by the finger. After detachment of the eschar, the sound parts being guarded by the application of wool dipped in carbonate of soda, plugs of wool moistened with alcoholic solution of bromine (one part of the latter to five of the former) were pressed against the diseased parts for five to ten minutes. He is of opinion that the solution of bromine attacks the new growth more energetically than the normal tissue, and in two cases of which he records the details there was every reason for believing that a permanent cure had been effected. (*Sitz.-Ber. der physic-med. Soc. zu Erlangen*, 1873.)

